# Exploring the Significance of Legal Status on Refugees’ and Asylum Seekers’ Access to Health Care in Germany—A Mixed-Method Study

**DOI:** 10.3389/ijph.2023.1605578

**Published:** 2023-06-21

**Authors:** Anna Christina Nowak, Oliver Razum, Claudia Hornberg

**Affiliations:** ^1^ School of Public Health, Bielefeld University, Bielefeld, Germany; ^2^ Department of Epidemiology & International Public Health, School of Public Health, Bielefeld University, Bielefeld, Germany; ^3^ Department of Sustainable Environmental Health Sciences, Medical School OWL, Bielefeld University, Bielefeld, Germany

**Keywords:** health care, utilization, refugee, mixed-methods study, postmigration stress, asylum seeker

## Abstract

**Objectives:** The study aims to investigate the significance of legal status for well-being and access to and use of needs-based health care by asylum seekers and refugees in Germany.

**Methods:** Using a mixed-method-design, we first conducted a cross-sectional study to explore access to health care and unmet needs of refugees and asylum seekers and legal status. Data were analysed using descriptive statistics. For the qualitative study a heterogeneous sample was recruited from the quantitative data. Interviews were analysed using a deductive-inductive approach.

**Results:** Quantitative results showed that health care utilisation was associated with an unsecure legal status but not with unmet care needs. The in-depth qualitative study revealed that the legal status determines experiences of structural violence that can negatively affect well-being and associated access to health care.

**Conclusion:** An insecure legal status can affect access to health care for refugees and asylum seekers. In order to improve health, changes in living conditions and the removal of access barriers are necessary.

## Introduction

In 2015 and 2016, Germany was one of the largest receiving countries for asylum seekers and refugees worldwide [1]. More than 1.1 million asylum seekers and refugees arrived in Germany, mainly from Syria, Afghanistan, and Iraq [2]. Providing health and social care posed major challenges for federal states, municipalities and medical staff in Germany which leads to unequal (health) care for refugees and asylum seekers [3].

Migration, whether voluntary or involuntary, can elicit positive as well as negative health consequences [4]. The life course approach shows that individual, environmental, and contextual exposures impact health in countries of origin, are influential during migration and in the receiving countries, and can even have a health effect on the subsequent generation [4]. Refugees and asylum seekers, in particular, are exposed to health burdens before, during, and after their forced migration [5, 6]. In addition to experiences of direct physical violence, structural violence in host countries plays an important role in establishing inequalities between population subgroups. Structural violence is characterised by violent acts without a direct perpetrator or whose perpetrators are hidden under powerful institutions [7]. Structural violence leads to inequalities, exploitation, deprivation, and unequal rights through unequal power relations [8]. Structural violence is determined by avoidable restrictions for population subgroups that prevent them from meeting their basic needs and achieving the quality of life that would otherwise be possible [7]. For example, two Dutch studies [9, 10] have shown that asylum seekers suffered more often from depression, anxiety, and posttraumatic disorders than refugees with a regular residency status.

We define asylum seekers as people whose asylum procedures have not yet been completed or who have an uncertain residence status (e.g., a tolerance permit according to the German Asylum Act (“Duldung”)). Refugees are people who have long-term permission to stay in Germany under either subsidiary protection or the Geneva Convention, or are eligible for asylum according to Germany’s constitutional law (Grundgesetz).

One form of structural violence in Germany is the entitlement restriction asylum seekers face in health care, despite increased health risks. According to the asylum seekers’ benefit act, asylum seekers are only entitled to treatment for acute illnesses, pain, and pregnancy-related conditions in the first 18 months [11, 12]. The treatment of chronic and mental illness is not routinely covered. Hence, asylum seekers in Germany experience inequalities in access to care [13], and often have unmet medical needs [14]. Even if regular access to health care is granted, cultural, language, and structural barriers remain, such as lack of funding of interpreters [15–17]. Further research is needed to understand the role of legal status as a form of structural violence in access to, and utilisation of, the healthcare system [18]. We want to elicit from the perspective of RAS [19] which entitlements and restrictions are associated with the legal status that can have a negative impact on well-being and health care. Therefore, the study aims to answer the following research questions 1): What role does the legal status play for health and well-being of refugees and asylum seekers in Germany? 2) How does the legal status influence access to, and utilisation of, outpatient health care among refugees and asylum seekers?

## Methods

### Study Design

We conducted a mixed-method study using an in-depth sequential design in which the qualitative results helped to explain quantitative data [20]. Quantitative and qualitative data were linked during data collection and data analysis. First, an interviewer team interviewed refugees and asylum seekers living in collective accommodations and communal shelters with a questionnaire. Then, problem-centred interviews in a sub-sample were conducted to gain a deeper understanding of the quantitative data. Participants were informed about the study objectives in advance and were given the possibility to ask questions about the procedure before, during and after the interview. Ethical approval for the study and written informed consent from each participant were obtained.

### Quantitative Approach

#### Procedure

In the quantitative, cross-sectional design, a trained team interviewed refugees and asylum seekers in shared accommodations and communal shelters from February 2018 to August 2018 in one region in North Rhine Westphalia, Germany. Approximately 800 RAS were eligible for participation as many recognized refugees were already housed in their own flats. A paper-assisted personal interview approach was used. Face-to-face interviews in native languages of participants were conducted. Our interviewer team consisted of 12 men and 1 woman. 9 spoke Arabic fluently, 4 Kurmancî and 3 Farsi. They were prepared for their task by an interviewer training module. The focus of the training was on conducting interviews, dealing with (psychological) emergency situations and translating questionnaires. Refugees and asylum seekers were eligible if they were older than 18 years and spoke Arabic, Kurdish, Farsi, English, or German. We chose these languages because between 2015 and 2018 nearly 33% of the 1.57 million asylum applications were Syrians, 11.7% Afghans, 10.4% Iraqis and around 3% Iranian [21]. During this period, between 70%–75% of entrants were under 30 years of age and between 60%–65% of first-time applicants were male. The project team recruited a convenience sample during seven information events in the shared accommodations centres, by direct approach by knocking on doors or by referral. The participants could choose the location for the interview. They usually took place in safe spaces in the shared accommodations or in the flats of the interviewees.

#### Measures

Sociodemographic characteristics included, gender, age, country of origin, country of refuge, education, professional background and legal status.

The number of contacts with the ambulatory (medical) health care system served to record access to medical services. We used and slightly modified parts of the “Health Questionnaire 18–64 Years of the Study on Adult Health in Germany” of the Robert Koch-Institute (DEGS1-RKI). For further analysis, we have formed a summed index by adding up the number of contacts with health care providers. Based on the findings of a published study [22], we designed a questionnaire to assess barriers in the provision of care and thus inequal access to health care. To assess unmet needs, participants were asked whether they had ever needed medical help in Germany. If this was answered affirmatively, we inquired whether the respondent had received help. If the interviewees affirmed both questions, we asked about barriers to accessing health care by formulating statements and asked for agreement and disagreement on a 5-point Likert scale (strongly disagree to strongly agree; or not at all true to very true). The questionnaire on barriers included lack of information about the German health care system, language difficulties, the feeling of not being taken seriously in health care, the search for help in the social surrounding, fear of exclusion, experiences of discrimination, transport difficulties, and structural barriers such as the lack of treatment vouchers, a lack of authorisation for medical treatment or the fear of legal consequences under residence law. An example of a question was “I’ve found it hard to use the range of services available in the German healthcare system because I can’t get a medical voucher.” The entire questionnaire was pre-tested in the target group for content and language comprehensibility. No validity or reliability tests were carried out.

All questionnaires were professionally translated and then back-translated by our interviewer team for quality control. The interviewer team discussed discrepancies in small groups and the project team adapted the questionnaires if necessary.

#### Statistical Analysis

Descriptive statistics were used to describe sociodemographic variables, legal status, health care usage and unmet needs. Means and standard deviations were determined were possible. We used cross tables to assess differences in health care utilisation and unmet needs between refugees with permission to stay (refugees or persons under subsidiary protection) or an unsecure legal status (in asylum procedure, deportation ban, tolerance permit). SPSS version 28 for Windows was used for all calculations.

### Qualitative Approach

We used the qualitative approach to interpret quantitative results in more detail.

#### Procedure

We used a qualitative sampling plan [23] to systematically select potential interviewees in order to consolidate the findings from the quantitative survey and to include heterogenous cases. We included male and female refugees and asylum seekers from different age groups, persons reporting good, medium and poor health, people with high, medium and low health care utilisation, people reporting unmet care needs and did not receive help and individuals with high, medium and low exposure to postmigration stressors. The first author identified 50 potential participants in the quantitative data set. For 18 persons, incomplete contact data or no informed consent was available. From the 50 identified people, 19 took part in the problem-centred interviews between August and October 2018. One person dropped out during the interview because he disagreed with recording and transcription of the interview (response rate: 56%). The first author developed an interview guide that contained narrative and open-ended questions on health status, experiences with health care in Germany and the current living situation. The first author (female, research associate, PhD student) interviewed participants individually at a location of their choice, usually in the participants’ accommodation. Thus, other family members were sometimes present and provided additional information. Field notes were written after the interview to reflect on the interview situation and to document special incidents. The interviews lasted on average 1 hour.

Qualified interpreters provided conversational interpreting during the interviews. All but two interviews were conducted in the participants’ mother tongue. One interview was conducted in German, one interview was conducted partly in English and partly in Arabic. All interviews were audio-recorded and transcribed verbatim in the native language of the interviewees and in German. For quality reasons, the native-language parts were translated again later. Due to time and language reasons, transcripts were not returned to participants for validation.

#### Data Analysis

We used a deductive-inductive content analysis approach [24]. In the first step, the first author prepared case summaries of the individual interview partners that were oriented towards the central themes of the interview guide (current health issues, experiences with medical care and current life situation). In the second step, thematic main categories were deductively developed. They were informed by the research question and the interview guide. The first author defined the categories, discussed them within a qualitative research group, and coded the entire interview material alone. Subsequently, the first author created subcategories using open coding [25]. In this way, we could identify categories that derived from the data and had a therefore a high relevance for the interview partners but were not captured by the interview guide. It has been shown that resources, especially the legal status, play an important role for RAS in the foreign living environment. Finally, the first author coded the interview material with the subcategories using MAXQDA Version 20.

## Results

In total, *n* = 198 people were interviewed quantitatively (response rate: 24.75%). The population characteristics are presented in Table 1. Their mean age was 33 years (SD: 11.02). 75.8% were male, originating mainly from Syria (41.4%) and Iraq (26.3%). 46.5% of respondents speak Arabic, followed by Kurdish (34.4%) and Farsi (12.6%). The mean duration of stay in Germany was 28.2 months (SD: 11.25). In the qualitative subsample, we interviewed 11 men and 7 women. The interviewees were older on average than in the quantitative study part with a mean age of 38.9 years (SD: 12.01). In total, 8 interviews were conducted in Arabic, 2 in Farsi, 6 in Kurdish, 1 in German and 1 in English and Arabic. Most participants were from Iraq (*n* = 8, 44.4%), followed by Syria (*n* = 6, 33.3%), Iran (*n* = 2, 11.1%), Afghanistan (*n* = 1, 5.6%) and Lebanon (*n* = 1, 5.6%). Five participants (27.8%) indicated poor or very poor health, five people (27.8%) indicated average health and 8 people (44.4%) indicated good or very good health. On average, they had sought medical care 12 times in the last 12 months. The frequency of use ranged from 0 to 58 physician contacts. Two interview partners stated use of psychotherapeutic care.

**TABLE 1:** Sociodemographic characteristics of participants (FlüGe Health Study, Germany, 2018).

	Quantitative study	Qualitative study
Age	Mean: 33.3 years (SD: 11.02)	Mean: 38.94 years (SD: 12.01)
Sex	23.7% Female	38.9% Female
	75.8% Male	61.1% Male
	(*n* = 1 missing value)	
Countries of Origin	41.4% Syria	44.4% Iraq
	26.3% Iraq	33.3% Syria
	9.6% Afghanistan	11.1% Iran
	6.6% Iran	5.6% Afghanistan and Lebanon
Housing Situation	39.4% Flat	22.2% Flat
	57.5% Shared Accommodation	78.8% Shared accommodation
	(*n* = 6 missing values)	
Residence Status	22.7% in the asylum procedure	29.4% in the asylum procedure
	42.9% recognized as refugee	33.4% recognized as refugee
	19.9% subsidiary protection	16.7% subsidiary protection
	11.6% tolerance permit or deportation ban	16.7% tolerance permit or deportation ban
	(*n* = 7 missing values)	

In the quantitative study, almost 90% of the respondents had visited ambulatory health care facilities since their arrival. They used health care on average 9.47 (SD: 12.12) times in the last 12 months. Family doctors (65.2%) and dentists (65.2%) had been consulted by the largest proportion of participants. 167 participants (84.8%) had a subjective need for medical care since their arrival in Germany. About one fifth of participants (20.7%) reported unmet needs. The most frequently mentioned underlying reasons included language difficulties (*n* = 28), lack of information (*n* = 27), a feeling of not being taken seriously (*n* = 24), having sought help in the social environment (*n* = 16), as well as a lack of authorisation for treatment (*n* = 14) or lack of treatment vouchers (*n* = 11). Nearly half (*n* = 8) of our qualitative interviewees reported unmet care needs due to lack of insurance and lack of authorisation for treatment, short duration of stay in accommodation facilities, missing social support or an unsecure living situation. In the interviews was pointed out that the interviewees experienced transitional living situations that were significantly influenced by residence status and the associated privileges and disadvantages. This is shown by one male interview partner in detail (I9, 128):

“The situation has changed from all aspects. We were in difficulties. The home was mixed. The food did not taste good. But that was only for six months. After that we were sent to flats. The children went to school. We have health insurance. We are getting better day by day and month by month.”

These changes are also evident in health care. At the beginning of their stay in Germany, the interviewees attended the medical doctors who worked in the central accommodation facilities. Regular access, however, often did not take place, despite a high need for it. One interview partner mentioned the “*transfers*” (I2, 201) from one shelter to another as a limiting factor for accessing regular care. Six interview partners experienced the lack of an electronic health card during the asylum procedure as a barrier to accessing full health care. Structural and bureaucratic barriers create a sense of powerlessness. Bureaucratic concessions, such as the acquisition of an electronic health insurance card therefore led to experiences of accessibility. One interviewee described how he “*demanded*” to be provided with prosthetic legs, but was refused by the social welfare office because it was not a “*life-threatening*” illness (I11, 172). As a result, the participant was restricted in his social participation (I11, 176).

“I saw the healthy people. People standing and walking normally. I wish that too. I couldn’t even go to the toilet by myself. I felt ashamed. I felt my weakness […]”

However, the participant knew that access to health care was only a “*question of time*” (I11, 170).

Four interviewees got stuck in this transitional phase. This was particularly evident in the absence of a residence permit or missing social support. In these cases, the interviewees mainly reported unmet care needs. One interviewee even compared medical care with the asylum procedure (I7, 102 ff.):

“The doctor writes down all these things (…). Everything that has happened to me. Like the judge”

In this case, the residence status also conditions the attendances of health care. Since, for example, psychiatric expert opinions are sometimes necessary for a residence status, the interview partners report that they visit (psychiatric) health care particularly often. This can be one explanation for the outliers of health care usage within the quantitative data. Especially when unmet needs arise, health care is frequented often.

At the time of the survey, 22.7% of the respondents were currently undergoing the asylum procedure; 42.9% had already been recognised, as either refugees or asylum seekers; and 11.6% had a legally uncertain residence situation. Our qualitative data show, that the significance of the residence status depends on whether persons entered Germany alone or via family reunification. Women who have joined their husbands reported hardly few experiences with dealing with authorities and the asylum procedure. One male interviewee explained that he had already gained a lot of experience with dealing with the authorities and had even helped other asylum seekers at the social welfare office to be prepared for his family to join him (I5, 136).

A secure residence status is attributed existential significance for psycho-social wellbeing by the interviewees even if the asylum procedures are already completed. One female interviewee describes:

“Since I came to Germany and until I got a residence permit, I felt a lot of psychological stress. I even brought them all my documents and told them that I didn’t want to stay in Germany anymore” (I3, 26)

The constant and recurring disclosure of the private situation during the asylum procedure, but also during other visits to the authorities, is also experienced as a burden:

“You are always under pressure. You always have to tell, always about private things”, describes one female interviewee (I18, 39)

The descriptive analysis showed that persons with an uncertain residence status (Mean = 14.81) sought health care twice as often as persons who had a permission to stay (Mean = 7.27). No descriptive differences for unmet care needs were found. 28 participants with a secure legal status (total *n* = 106, 26.4%) stated that they had ever needed medical help but had not received it, compared to 12 persons with an insecure legal status (total *n* = 49, 24.5%). The qualitative results indicated that the fear of non-recognition of documents, the fear of deportation, and the fear or the suspension of family reunification were experienced as “*paralysing” (*I19, 10) structures because they cannot be influenced. One interviewee (I19, 87) described how the psychological suffering caused a somatic reaction leading to health care usage:

“My eyelid trembles and then closes down. My eye problem gets worse with sadness and stress”

Interviewees experienced different privileges through a long-term permission to stay, e.g., in access to the labour market or finding an apartment. If residence is denied, social withdrawal may result. One male interviewee describes fear of going to school (I9, 15), fear of the police (I9, 303), fear of going to the court (I9, 128) and fear of using health care (I9, 79). A rejection decision could foster vicious cycles that led to social withdrawal and a fearful living situation. Health care was then no longer sought—despite a high need for (psychological) care (I9, 156):

“When I arrived in Germany, my condition was normal. I went before the court and told my story. The court rejected my asylum application. The lawyer told me the reason was that my city of origin was a safe city. After the court rejected it, my health began to deteriorate […]. I have not gone to any doctor. I am afraid to go. I have a psychological problem"

## Discussion

We found that well-being, access to health care, and use of the health care system by RAS is influenced by their residence status. Without a long-term residence permit, RAS a) do not perceive sufficient opportunities for their personal and health development; b) are excluded from social participation due to access barriers and entitlement restrictions in health care; and c) can therefore be conceived as victims of structural violence according to Galtung [8]. Krause [26] has shown that RAS experience structural violence through discriminatory practice in access to cultural, financial, social and health benefits. Our study shows that the granting of a regular residence status is of particular importance because it entails privileges and lowers access barriers to social and health services. Interviewees named formal and legal restrictions, such as the lack of an electronic health card or the lack of financing interpreters, as challenges. We interpret them as mechanisms of (presumably politically intended) structural violence in access to health care. Limited entitlements to health care and the issuing of treatment vouchers created access barriers for asylum seekers and bureaucratic hurdles for medical staff [27, 28]. Providing an electronic health card early in the asylum procedure and allowing direct access to the health care system without entitlement restrictions would remove access barriers [29] and lower costs [30]. The same applies to the provision of interpreters [31]. Apart from unmet care needs, the qualitative results suggest that limited access to health care prevents social participation, with negative consequences for integration efforts and psychological well-being of individuals.

Nevertheless, the frequency of outpatient health care utilization among RAS was high and did not differ from the German population [32]. This can be partly explained by the long duration of stay at the time of the survey. At the same time, the quantitative analysis shows that an insecure residence status is deterministic for an above-average use of healthcare. We found that persons with an uncertain legal status sought health care twice as often as person with a legal entitlement. This underlines the importance of the residence status and accompanying mechanisms of structural violence for health care usage and well-being. Furthermore, the qualitative results clearly show the entitlement restrictions during the first 18 months of the asylum procedure were perceived as obstacles in access health care. Even though health care is sought often, a large proportion of the respondents reported unmet needs. This was also shown by Schneider et al. [13] and Bozorgmehr et al. [14]. At the same time, as also described in a qualitative study by Schneider et al. [33], health needs and health care usage can be partly superimposed by reception conditions or experiences with the authorities. Although negative effects on psychosocial well-being due to an uncertain residence status or negative experiences with authorities were reported in the qualitative interview study, health care was not sought by the interviewees. In some cases, this uncertainty initially leads to frequent visits to health care, which, however, is not experienced as needs-based. The result can be withdrawals from health care. The existing structural violence in the reception situation can thus negatively influence health and perpetuate mechanisms of exclusion.

### Strengths and Limitations

Our study has a methodological strength, namely the linkage of quantitative and qualitative data. But there are also limitations. First, the study is based on convenience sampling, so we cannot exclude that people who use health care more or less than average preferentially participated in the survey. Hence, results should be interpreted with caution and cannot be generalised. Second due to the cross-sectional study design, it is not possible to conclude how changes in residence status affect healthcare access. Third, questions on barriers in access to health care were only asked to participants reporting unmet needs. However, asking these questions to all respondents may not have elicited additional information as all additional access barriers would by definition have been surmounted.

Finally, we tried to reduce interviewer and interpreter bias in both the quantitative and the qualitative survey but cannot exclude that it may have been present. In the interpreter-assisted interviewing it was difficult for the researchers to ask more in-depth questions at certain points due to not speaking the language of participants. We used the translated transcripts for analysis to reduce translation errors during the interviews. Moreover, it was not always possible to conduct individual interviews due to crowded conditions in accommodation facilities. This may have affected the responses of interviewees.

### Conclusion

The legal status is experienced of crucial importance for well-being and health care usage of RAS in Germany. Strategies to improve the situation could focus on alleviating stress in this group, as well as on removing structural access barriers in health services to avoid structural violence. Intervention studies need to assess which approach is more effective.
